# *Candida auris* in a tertiary healthcare setting in south India: A case series

**DOI:** 10.22034/CMM.2024.345186.1502

**Published:** 2023-12

**Authors:** Asem Ali Ashraf, Rohan Steve Pinto, Vimal Kumar Karnaker

**Affiliations:** 1 Department of Microbiology, Nitte (Deemed to be University), KS Hegde Medical Academy (KSHEMA), Deralakatte, Mangalore, Karnataka, India; 2 Intern, B.Sc. in Medical Laboratory Technology, Nitte (Deemed to be University), KS Hegde Medical Academy (KSHEMA), Deralakatte, Mangalore, Karnataka, India

**Keywords:** Antifungal agents, *Candida auris*, Identification, Infections, MALDI-ToF

## Abstract

*Candida* species can produce a variety of clinical manifestations, and several non-*albicans* species of *Candida*, including *Candida auris*, have been linked to the rise of invasive fungal infections with high rates of treatment failure. Nosocomial outbreaks and high mortality rates in healthcare institutions across the globe have been associated with *C. auris*, an emerging infectious yeast that was initially discovered in the ear canal of an elderly Japanese patient in 2009. The fact that *C. auris* has been found on six continents after it was initially isolated has raised serious concerns among scientists and healthcare practitioners.

At present, healthcare facilities lack defined protocols for the effective prevention and control of *C. auris* infections, as well as appropriate treatment alternatives. This leads to frequent therapeutic failures and complicates the eradication of *C. auris* infection in healthcare facilities. Studies on *C. auris* in South India are often limited, and healthcare workers urgently need to be made aware of infections caused by it in order to assess its impact and possible implications for the healthcare system. This study aimed to report seven patients hospitalized in our center who developed *C. auris* infections with varying clinical manifestations.

## Introduction

*Candida* species (spp.) can produce a wide range of clinical symptoms, such as superficial infections, intra-abdominal candidiasis, deep-seated candidiasis, and bloodstream infections[ 1 ]. Infections due to *Candida* spp. have grown significantly during the past few decades, primarily due to the rising number of invasive procedures, the injudicious use of broad-spectrum antibiotics, and the immunocompromised state of critically ill patients. Several non-*albicans* spp. of *Candida*, including *Candida tropicalis*, *Candida glabrata*, *Candida parapsilosis*, and *Candida krusei*, are responsible for the rising incidence of invasive fungal infections with high rates of therapeutic failure, despite the fact that *Candida albicans* is the most common cause of hospital-acquired fungal infections [ 2 ]. 

A couple of clinical alerts were issued by the Centers for Disease Control and Prevention (CDC) in 2016 and 2017 to alert people about the development of *Candida auris* (*C. auris*) infections [ 3 ]. *C. auris* and other multidrug-resistant nosocomial pathogens were once again listed as the main urgent threats in the 2019 Report of the CDC on Urgent Threats [ 4 ]. The Indian Institute of Medical Research published an advice note to guarantee that this species is actively monitored in Indian hospitals [ 5 ]. More and more cases of *C. auris* have been reported from various nations; therefore, clinicians, infection prevention and control practitioners, as well as public health officials, should be aware of how to mitigate the threat posed by this pathogen since it is critical to take preventative control measures to halt its spread. 

Studies on the drug resistance profile of *C. auris* in South India are often limited, and healthcare workers urgently need to be made aware of infections caused by this pathogen in order to assess its impact and possible implications for the healthcare system. This study aims to report seven patients hospitalized in our center who developed *C. auris* infections. 

## Materials and Methods

### Study Design and Setting

This retrospective, single-center study was conducted at a 1,000-bed tertiary healthcare teaching hospital in southwest coastal Karnataka, India, from January 01, 2020, to June 30, 2023. All patients admitted to any ward and the Intensive Care Unit (ICU) of this hospital with isolation of *C. auris* were included in the research. This study was approved by the Institutional Ethics Committee (INST.EC/EC/116/2023).

### Microbiological Analysis

The growth of *C. auris* obtained on 5% Sheep Blood agar was tiny white opaque, non-hemolytic colonies. The Gram stain revealed gram-positive budding yeast cells, and the respective colonies were sub-cultured on HiChrome agar (HiMedia), revealing colorless colonies in 24 h that became pink-purplish on further incubation. C. auris was further identified in clinical specimens with the VITEK 2 Compact System (bioMérieux, Marcy L’Etoile, France), an automatic identification system, and a matrix-assisted laser desorption/ ionization-time of flight (MALDI-ToF)-based automated bacterial identification system (bioMérieux, France).

Antifungal susceptibility testing (AFST) for amphotericin (AMB), fluconazole (FLU), voriconazole (VRC), caspofungin (CAS), micafungin (MFG), and flucytosine (FC) was carried out using an automated turbidimetric system (Vitek 2 AST YS08, bioMérieux, France). 

The drug concentration ranges were 0.25-16 mg/mL for AMB, 0.25-4 mg/mL for CAS, 1-64 mg/mL for FLU and FC, 0.06-4 mg/mL for MFG, and 0.125-16 mg/mL for VRC. Since no species-specific susceptibility breakpoints are currently available for *C. auris*, AFST results were interpreted according to the tentative breakpoints proposed by the US CDC [ 6 ].

### Data Collected for the Analysis

The demographic characteristics, microbiology laboratory results, and clinical information from electronic medical records of all positively identified *C. auris* cases from January 01, 2020, to June 30, 2023, were reviewed and recorded.

## Case Reports

The clinical features and risk factors of patients with *C. auris* infections in 7 cases are presented in Table 1, while Table 2 displays the antifungal susceptibility of isolates in terms of minimum inhibitory concentration.

**Table 1:** Clinical features and risk factors of patients with Candida auris infections

	Case 1	Case 2	Case 3	Case 4	Case 5	Case 6	Case 7
Demographics	67 years old, Male	65 years old, Male	23 years old, Female	64 years old, Male	61 years old, Male	63 years old, Male	71 years old, Male
Underlying disease	Type 2 diabetes mellitus	Type 2 diabetes mellitus	-	Hypertension	-	Type 2 diabetes mellitus	Hypertension
	Hypertension	Hypertension		Ischaemic heart disease		Hypertension	Ischaemic heart disease
		Ischaemic heart disease		Chronic kidney disease		Ischaemic heart disease	Chronic obstructive pulmonary disease
		Old cerebrovascular accident		Ischaemic heart disease			
Diagnosis	Pneumonia	Pneumonia	Extrapulmonary tuberculosis (pleural bilateral pneumothorax)	Sepsis with septic shock	Grade IV pressure sore over the lower back region	AKI	Pneumonia
	ARDS	ARDS	Stress cardiomyopathy	CKD (S/P 3 cycles)		CKD	Sepsis with septic shock
	Sepsis with Septic shock in MODS	AKI		Fracture-left neck of femur		Right foot gangrene	Acute cerebrovascular accident
	AKI	Lumbar spondylosis				Esophageal candidiasis	
	HD (S/P 5 cycles)						
Type of clinical sample	Blood	Catheterized urine	Pus (intercostal drainage tube site)	Blood	Midstream urine	Blood	Blood
Ward/ICU	ICU	ICU	GW	ICU	GW	ICU	ICU
Duration of hospital stay (days)	28	28	81	18	20	80	33
Outcome	DAMA	Survived	Survived	DAMA	Survived	Survived	Expired
Type of candidiasis	Invasive candidiasis	Non-invasive candidiasis	Non-invasive candidiasis	Invasive candidiasis	Non-invasive candidiasis	Invasive candidiasis	Invasive candidiasis
IMV	Yes	Yes	Yes	Yes	Yes	Yes	Yes
CVC	Yes	Yes	No	Yes	No	Yes	Yes
BSA	Yes	Yes	Yes	Yes	Yes	Yes	Yes
AFT	No	No	No	No	No	No	No
Surg	No	No	No	Yes	No	Yes	No
TPN	Yes	Yes	No	Yes	No	Yes	Yes
Uri Cat	Yes	Yes	No	Yes	No	Yes	Yes
Candiduria	No	Yes	No	No	Yes	No	No

ARDS: Acute Respiratory Distress Syndrome, AKI: Acute Kidney Injury, AFT: History of anti-fungal therapy, BSA: Broad-spectrum antibiotics, CVC: Presence of Central Venous Catheter, CKD: Chronic Kidney Disease, DAMA: Discharged against Medical Advice, GW: General Ward, HD: Haemodialysis, ICU: Intensive Care Unit, IMV: Invasive Mechanical Ventilation, MODS: Multiple Organ Dysfunction Syndrome, S/P: Status Post, Surg: Surgery within 30 days, TPN: Total Parenteral Nutrition, Uri Cat: Indwelling Urinary Catheter

**Table 2:** Antifungal susceptibility of isolates in minimum inhibitory concentration (MIC (μg/mL))

Case no	FLU	VRC	FC	AMB	CAS	MFG	Antifungal medication provided for the patient
1	SDD (4)	R (2)	R (≥64)	R (2)	S (0.25)	S (0.12)	The patient was discharged against medical advice; hence, no antifungal therapy was initiated on the patient.
2	R (8)	R (1)	R (≥64)	R (8)	S (0.25)	S (≤0.06)	No antifungal therapy was recommended as there was no evidence of infection.
3	R (32)	R (1)	R (≥64)	R (8)	S (0.25)	S (≤0.06)	No antifungal therapy was recommended as there was no evidence of infection.
4	R (8)	R (2)	R (≥64)	R (2)	S (0.25)	S (0.12)	The patient was discharged against medical advice; hence, no antifungal therapy was initiated on the patient.
5	R (16)	R (2)	R (≥64)	R (8)	S (0.25)	S (0.12)	No antifungal therapy was recommended as there was no evidence of infection.
6	R (32)	R (2)	R (≥64)	S (0.5)	S (0.25)	S (0.12)	The patient was started on injection of Caspofungin 50 mg (intravenous) OD with a loading dose of 70 mg IV, in view of candidemia. IV Caspofungin was provided for a total of 18 days.
7	R (32)	R (2)	R (≥64)	R (8)	S (0.25)	S (0.12)	The patient was started on injection of Caspofungin 50 mg (intravenous) OD with a loading dose of 70 mg IV, in view of candidemia. IV Caspofungin was given for a total of 11 days.

AMB: Amphotericin; CAS: Caspofungin; FC: Flucytosine; FLU: Fluconazole; MFG: Micafungin; MIC: Minimum inhibitory concentration; SDD: Susceptible-dose dependent; VRC: Voriconazole; OD: Once a day

### Case 1

The patient had developed a sudden-onset fever, associated with dry coughs and breathlessness, for five days. In view of low oxygen saturation and tachypnea, the patient was put on non-invasive ventilation (NIV) and managed conservatively with IV fluids, IV antibiotics, antivirals, anti-coagulants, and steroids. During the hospital stay, the condition deteriorated, and thus the patient was intubated and put on mechanical ventilation. Deranged renal function tests and decreased urine output were noted, and hemodialysis was advised. Due to low blood pressure, the patient was started on multiple inotropes. However, in view of financial constraints, the family members of the patient were not willing to pursue further clinical management; therefore, the patient was discharged against medical advice after 28 days of hospital stay.

### Case 2

The patient had developed a sudden-onset fever, associated with chills and coughs with expectoration, for three days. Additionally, the patient also complained of sudden-onset breathlessness for one day, which was progressive in nature. He was put on NIV and eventually intubated in view of respiratory distress. He was started on broad-spectrum antibiotics, antivirals, IV steroids, and anticoagulation. 

Oxygen support was gradually reduced, and the ventilator was tapered off. He was extubated, his saturation levels were regularly monitored, and the oxygen was gradually reduced. On the day of discharge, he had a sudden onset of hypotension and desaturation and was readmitted to the ICU. He was started on inotropes, and a cardiology opinion was sought in view of elevated cardiac markers. An electrocardiogram (ECG) showed non-ST-elevation myocardial infarction; therefore, a coronary angiogram was advised at a later date once the patient was clinically stable. Gradually, his inotropes and oxygen support were tapered, and as the clinical parameters of the patient improved, he was moved to the general ward and was eventually discharged in stable condition after 28 days of hospital stay. 

### Case 3

The patient had developed coughs associated with breathlessness for two months, which gradually became progressive and exacerbated for one week. The patient also complained of fever for one week with a rise in temperature in the evenings. Additionally, the patient recorded a weight loss of 1 kg in one month. Upon evaluation, the patient was diagnosed with pneumothorax. The inter-costal drainage (ICD) insertion was performed on the right side. The patient was noted to have increased sweating and tachypnoea on the following day and was found to have left-sided tension pneumothorax. Consequently, the ICD insertion was performed on the left side. 

The patient was electively intubated in view of increasing breathlessness and tachypnea. Pleural fluid was sent for acid-fast bacilli staining and was found to be positive, for which the patient was subsequently started on anti-tubercular therapy. As the condition of the patient started to clinically improve, she was eventually extubated, and both her ICD tubes were removed. However, the patient started developing repeated episodes of fever, and the pleural fluid culture showed *Pseudomonas aeruginosa* sensitive to ciprofloxacin. She was started on tab ciprofloxacin, and the total counts started reducing subsequently. 

The patient developed sudden-onset breathlessness and was shifted to the High Dependency Unit. Abnormal ECG changes were observed, which showed features suggestive of stress cardiomyopathy. As a consequence, she was put on a non-rebreathing mask, with the right ICD tube in situ connected to wall suction. The patient’s condition became better, and she maintained saturation in room air. The patient was eventually shifted out to wards, and as her clinical parameters started to gradually improve, her ICD tube was removed, and she was symptomatically better. She was discharged in stable condition after 81 days of hospital stay.

### Case 4

The patient was admitted in view of altered sensorium for two days. He was started on IV antibiotics due to infective endocarditis. An orthopedic opinion was sought in view of the left neck of the femur fracture, and the patient was planned for under high-risk circumstances. However, his family members were not willing to do the surgery. He was shifted to the ICU in view of further worsening of the sensorium, desaturation, and hypotension. He was started on inotropes and other supportive measures. The guarded prognosis was explained to family members of the patient; however, they were not willing to continue further treatment. Therefore, the patient was discharged against medical advice after 18 days of hospital stay.

### Case 5

The patient complained of pain and pus discharge from the back for one month. He had had an alleged history of a fall a couple of months earlier, as a result of which he had sustained an injury to the lower back and left leg and was diagnosed with L4 and L5 dislocations with a complete spinal cord injury. He underwent an anterior cervical discectomy and fusion of L4 and L5 vertebrae. Following clinical examination, the patient was diagnosed with Grade IV pressure sores in the lower back region. The patient underwent wound debridement and local flap closure under general anesthesia. It should be mentioned that the postoperative period was uneventful. The patient has been doing well symptomatically and was discharged in stable condition after 19 days of hospital stay.

### Case 6

The patient with an alleged history of trauma to the leg had started developing an ulcer over the right third toe, associated with gangrenous changes (disarticulation of the second and third toes), followed by the formation of an ulcer extending over the dorsum and plantar aspect for one month. The patient was being treated for it in a local hospital, where he developed sudden-onset breathlessness associated with scanty mucoid coughs with expectoration and altered sensorium.

The patient was evaluated and diagnosed with severe left ventricular dysfunction with acute pulmonary edema. As a consequence, the patient was intubated in view of respiratory distress and treated with IV antibiotics, diuretics, and other supportive medications. Gradually, as the general condition of the patient improved, he was extubated and shifted to the general ward. A surgical opinion was sought in view of the right foot gangrene, and the patient underwent debridement followed by regular dressing. He had developed pulmonary edema once again and was re-shifted to the ICU, where he was intubated and given IV diuretics. 

As his clinical condition improved, the patient was extubated and conservatively managed on antihy-pertensives, antiplatelets, diuretics, and antibiotics. Regular dressing was performed, the pus was sent for culture sensitivity, and antibiotics were administered accordingly. A surgery review was conducted in view of gangrene, and skin grafting was advised. Following the aforementioned surgical procedure, the patient did well symptomatically, and after 80 days of his overall hospital stay, he was discharged in stable condition. 

### Case 7

The patient was brought to the casualty complaining of a low-grade fever for four days, associated with cough and hemoptysis. The patient also had breathlessness for two days with no diurnal or postural variations. He was admitted to a local hospital and managed with antibiotics. 

During his hospital stay, he developed right-sided weakness and slurring of speech and was diagnosed with an acute infarct over the left capsuloganglionic region (left temporal lobe), after which he became drowsy and was suspected to have aspiration pneumonia. Therefore, he was referred to the present tertiary healthcare setting. 

The patient was admitted to the ward, and upon further clinical evaluation, he was diagnosed with bronchopneumonia with an acute exacerbation of chronic obstructive pulmonary disease and an acute cerebrovascular accident. In the ward, the patient had sudden desaturation and tachypnoea, and in view of the worsening Glasgow Coma Scale and desaturation, he was shifted to the ICU and intubated. Despite the medical intervention, the patient eventually succumbed to infection and died on the 33^rd^ day of his hospitalization. 

## Discussion

This study assessed the range of infections brought on by *C. auris* in both immunocompromised and immunocompetent patients who had previously undergone surgery, received broad-spectrum antibiotic treatment, had urinary and central venous catheters inserted, and were admitted to ICUs. 

Numerous invasive infections can occur in all age groups due to the multi-resistant nosocomial fungal pathogen *C. auris*, which is also linked to an elevated mortality rate [ 7 ]. A wide range of clinical symptoms have been documented, including meningitis, pericarditis, peritonitis, bloodstream infections, and urinary tract infections [ 8 , 9 ]. The length of time spent on mechanical ventilation, the use of total parenteral nutrition, central venous catheters, cross-transmission by medical staff, the use of broad-spectrum antibiotic therapy, and the use of corticosteroids are some of the known risk factors for invasive candidiasis. Additionally, some previous studies have indicated that prior fluoroquinolone or carbapenem usage is a strong independent predictor of candidemia [ 9 , 10 ].

The findings of the present study also revealed that *C. auris* was largely resistant to FLU, with a high minimum inhibitory concentration (MIC) value of 8-32 mg/mL in all seven patients. This is in contrast with the results of a study conducted by Mathur et al. (2018) [ 11 ], where 45% of *C. auris* isolates had lower MIC values for FLU, ranging from 0.05 to 4 mg/mL. Another study carried out by Chowdhary et al. (2018) analyzed 350 C. auris isolates collected from 10 different hospitals in India and reported that 90% of *C. auris* isolates were FLU-resistant (MICs 32 to ≥64 mg/L) [ 12 ], which is in concordance with the results of the present study. 

Since FLU is the most frequently used empirical antifungal medication in Indian hospitals, particularly in cases of unexplained fever or sepsis when the patient is not responding to antibiotics, this pattern of antifungal resistance is particularly concerning and disturbing. An alarming percentage of isolates (85%) exhibited elevated MICs (4-8 mg/mL) of amphotericin B detected by the VITEK 2 automated system, which is in agreement with the findings of the study performed by Chowdhary et al. (2018) [ 12 ]. Due to the concern of azole and amphotericin B resistance, echinocandins are advised as a first-line treatment and subject to anti-fungal susceptibility testing. 

It is noteworthy that we found all isolates of *C. auris* that were susceptible to CAS and MFG. This is in contrast with an analysis of 102 *C. auris* isolates conducted by Kathuria et al. (2015) from four different hospitals in India, where 37% of *C. auris* isolates showed reduced susceptibility to echinocandin class (MICs: 4 mg/mL for MFG and 2 mg/mL for CASP) [ 13 ]. Accurately identifying and testing for the antifungal susceptibility of *C. auris* is important for guiding therapy and evaluating prognosis in ICUs, where multidrug-resistant strains of the yeast are frequently prevalent.

If appropriate antifungal therapy is not provided in conjunction with other interventions, such as the removal of invasive colonization devices, invasive infections brought on by *C. auris* will likely result in increased mortality rates [ 10 ]. Given its pattern of resistance to antifungals, *C. auris* has emerged as a significant pathogen [ 10 , 14 ]. The use of broad-spectrum antibiotic medication is another important risk factor that is common among all the cases reported in these series and is well-known to be associated with invasive candidiasis. Although there is evidence that the use of piperacillin/tazobactam is a significant predisposing risk factor for systemic candidiasis, this risk is shared by several antibiotic classes [ 15 ]. 

In many microbiological laboratories, it can be difficult to confirm the diagnosis of *C. auris*. By using MALDI-ToF in this study, it was possible to confirm the presence of *C. auris* in our patients. This technique, which has become a standard for routine bacterial isolate identification in clinical microbiology laboratories worldwide, is now also essential for the accurate identification of *C. auris* isolates. This is crucial and ought to be taken into account if individuals exhibit symptoms and risk factors for a *C. auris* infection. Molecular characterization or protein profiling using MALDI-ToF is currently recommended by the World Health Organization to validate the identification of Candida spp. by reference or public health laboratories in order to exclude the possibility of *C. auris* infection. This is especially recommended in cases where other species of *Candida*, such as *Candida haemulonii*, *Candida guilliermondii*, *Candida famata*, *Candida sake*, *Rhodotorula glutinis*, and *Saccharomyces cerevisiae*, are identified [ 16 , 17 ].

To decrease the risk of transmission, healthcare personnel in acute care settings should use standard contact precautions. Given the high degree of antifungal resistance of *C. auris* isolates, as well as their ease of transmissibility in healthcare settings, it is imperative to quickly identify nosocomial outbreaks of the infection in order to implement infection control measures. Chlorhexidine-impregnated pads for central venous catheters, chlorhexidine washes, and mouthwashes are a few treatments that have demonstrated efficacy when used in bundles to stop the spread of *C. auris*. However, it is necessary to clarify how skin disinfection practices affect shedding and colonization. Since *C. auris* can live on a variety of surfaces, cleaning techniques and the application of disinfectants may be able to lower the risk of transmission [ 15 ].

## Conclusion

The multi-resistant nosocomial fungal pathogen *C. auris* can cause several invasive infections in all age groups and is associated with an increased mortality rate. For the purpose of guiding medication and assessing prognosis in ICUs, where multidrug-resistant strains of the pathogen are usually prominent, it is critical to accurately identify and test for the antifungal susceptibility of *C. auris*. Since *C. auris* isolates have a high level of resistance to antifungals and are highly transmissible in healthcare environments, it is critical to promptly detect nosocomial outbreaks of the illness to enable the implementation of infection control measures.
